# Augmentation in restless legs syndrome: an eye tracking study on emotion processing

**DOI:** 10.1002/acn3.51144

**Published:** 2020-08-12

**Authors:** Philipp Ellmerer, Beatrice Heim, Ambra Stefani, Marina Peball, Mario Werkmann, Evi Holzknecht, Melanie Bergmann, Elisabeth Brandauer, Martin Sojer, Laura Zamarian, Margarete Delazer, Klaus Seppi, Birgit Högl, Werner Poewe, Atbin Djamshidian

**Affiliations:** ^1^ Department of Neurology Medical University of Innsbruck Innsbruck Austria

## Abstract

**Objective:**

To assess emotional processing and alexithymia in patients with restless legs syndrome (RLS) with augmentation versus those who never had augmentation.

**Methods:**

We recruited 26 patients who had a history of augmentation (AUG), either current or past, 27 RLS patients treated with dopamine agonists who never had augmentation (RLS controls), and 21 healthy controls (HC). All participants were screened for impulse control disorders (ICDs). Alexithymia was assessed by means of the Toronto Alexithymia Scale – 20 (TAS‐20). Facial emotion recognition was tested through an eye‐tracking task. Furthermore, all participants performed neuropsychological tests assessing global cognitive status, impulsivity, anxiety, and depression.

**Results:**

ICD symptoms occurred more frequently in AUG patients than in RLS controls (*P* = 0.047). Patients with AUG scored higher on the TAS‐20 (*P* = 0.007) and the attentional subdomain of an impulsivity scale (BIS‐11; *P* = 0.015) compared to HC. Patients with AUG also performed worse on the facial emotion recognition task relative to RLS controls (*P* = 0.009) and HC (*P* = 0.003). We found a group difference for the time to first fixation and the fixation count in the mouth region (*P* = 0.019 and *P* = 0.021, respectively). There were no other differences in the eye tracking examination.

**Interpretation:**

This study showed evidence of poorer emotional processing in patients who had augmentation compared to RLS patients without augmentation and healthy controls. The altered exploration pattern of faces and the higher alexithymia scores suggest abnormalities in emotion processing in patients with augmentation.

## Introduction

Restless legs syndrome (RLS) is a common neurological sensorimotor disease, which is frequently associated with neuropsychiatric comorbidities such as anxiety and depression.^1^, ^2^, ^3^ Dopamine agonists (DA) are often used to alleviate the unpleasant motor symptoms.^4^ However, in some patients, these drugs induce unwanted side effects such as augmentation or impulse control disorders (ICDs).^5^ Neuroplastic changes caused by DA are believed to play a role in the development of ICDs^6^, ^7^ and have also been linked with augmentation.^8^, ^9^, ^10^ Surprisingly little is known about the neuropsychological profile of RLS patients with augmentation.^11^

Alexithymia is defined as a difficulty to identify, express, or describe own feelings and was significantly more present in patients with ICDs and substance abuse compared to healthy controls.^12^, ^13^, ^14^ Higher scores on an alexithymia scale in patients with Parkinson’s disease (PD) are thought to result from the alteration of dopaminergic neural pathways.^15^ The dysfunction of central dopaminergic signaling is also believed to play a role in the pathogenesis of RLS.^16^

In this study, we aimed to assess the effects of augmentation, which is believed to reflect a maladaptive response to sustained dopaminergic substitution in RLS, on alexithymia and recognition of different emotions. Since augmentation and ICD symptoms frequently co‐occur,^5^ we were especially interested in evaluating how RLS with current or a history of augmentation perform in tasks which have been proposed as risk factors for the development of ICDs.^13^, ^17^ Results were compared to patients without augmentation as well as to age‐, sex‐, and education‐matched healthy controls. We hypothesized that patients with current or past augmentation would have an impairment in emotional processing due to irreversible dopamine agonist induced neuroplastic changes in the cortico‐striatal network.

## Methods

The study was approved by the Research Ethics Committee of Innsbruck Medical University, Austria, before study initiation. All participants provided written informed consent according to the Declaration of Helsinki.

A total of 26 patients diagnosed with idiopathic RLS who had a history of or had current augmentation, 27 patients with idiopathic RLS treated with dopaminergic therapy who never had augmentation and 21 age‐, sex‐, and education‐matched healthy controls were prospectively enrolled between July 2017 and February 2019. Patients were recruited from the sleep disorders outpatient clinic and sleep laboratory of the Department of Neurology, Innsbruck Medical University. RLS diagnosis was made by board‐certified sleep specialists according to the International Restless Legs Syndrome Study Group criteria.^18^ We used previously published guidelines to define and classify augmentation.^19^ All patients were assessed by a board‐certified sleep specialist who carefully excluded potential confounding factors such as treatment tolerance, end‐of‐dose rebound, and fluctuations. Patients were screened for causes of secondary RLS, such as kidney failure,^20^ cardiac failure, and anemia.^21^ Furthermore, subjects with mild cognitive impairment (defined as a Mini Mental State examination (MMSE) score below a cut‐off score of 26 points), or psychiatric disorders, such as major depression, or apathy were excluded. Concomitant antidepressants were allowed if the dose was stable for 4 weeks prior to testing. Furthermore, patients with uncorrected visual impairment were excluded from participation. RLS patients were divided into two groups. Those who had a history of augmentation or had current augmentation (AUG) and those without augmentation or history of augmentation (RLS controls). Patients who partially met the criteria for augmentation according to Garcia‐Borreguero et al.^4^ were classified as subthreshold augmentation and also assigned in the augmented group. In a second step, patients with augmentation were subdivided into those with and without ICD symptoms. ICDs were assessed through a semistructural interview based on the Questionnaire for Impulsive‐Compulsive Disorders in Parkinson’s disease (QUIP).^22^ ICD symptoms were defined as a positive screen on the QUIP without causing a significant impairment as described earlier.^5^ These grouping criteria were predefined in advance of the first subject being screened for the study. A subgroup analysis of patients with current and past history of augmentation was performed. A total of 21 healthy participants from the same local area were included as healthy controls.

All participants were asked to perform a facial emotion recognition task consisting of 21 pictures selected from the Warsaw Set of Emotional Facial Expression Pictures (WSEFEP).^23^ Seven different emotions (neutral, joy, anger, disgust, fear, sadness, surprise) were repeated in total three times with different faces. The task was performed in a quiet room to avoid distractions. Pictures were presented on a 23” computer screen at a distance of 64 cm. After a decentralized fixation cross appeared on the screen for 1500 msec, patients were asked to freely explore a picture of a face for 5000 msec. Participants had to judge which of the seven possible emotions (neutral, joy, anger, disgust, fear, sadness, surprise) was presented. The task was administered always by the same researcher (PE). The answer was recorded by the experimenter. Eye movement data were collected through the Tobii TX‐300 eye tracker and the Tobii Pro Software (v1.83). Raw data were collected at 300 Hz sampling rate. A 9‐point calibration was performed before starting the facial emotion recognition task in order to assure gaze position accuracy under 0.4°. We used the predefined attentional velocity‐threshold identification filter implemented in Tobii Pro in order to detect fixations for our analysis. Three areas of interests (AOI; whole face, eye region, mouth region) were predefined for every stimulus. We used the parameters fixation duration relative to total duration, number of fixations per second and time to first fixation (TTFF) for every AOI.

Furthermore, participants were given the German version of the TAS‐20, which consists of the three subdomains F1 – “difficulty identifying emotions,” F2 – “difficulty describing emotions,” and F3 – “external oriented thinking.”^24^, ^25^ As previously described,^26^ total scores ≥ 61 on the TAS 20 were considered as definitive alexithymia. They also were tested with a short neuropsychological battery assessing global cognitive status (MMSE),^27^ impulsivity (German version of the Barrett Impulsiveness Scale‐11; BIS‐11),^28^, ^29^ anxiety and depression (German version of the Hospital Anxiety and Depression Scale; HADS‐D).^30^ For the patient groups only, levodopa equivalent daily doses (LEDD) were calculated as reported previously.^31^

All data analysis was carried out in R (V 3.6.3).^32^ Parametric or nonparametric tests were used for statistical analysis depending on the distribution and the scale type of the variables. If normality assumptions were met according to Shapiro–Wilk tests, parametric tests were applied. Otherwise, nonparametric tests were used. Quantitative values are given in mean ± SD or median and interquartile range depending on the distribution of data. All post hoc pairwise comparisons were carried out using Bonferroni correction. The level of significance was set at *P* < 0.05.

## Results

In total, 26 patients experienced symptoms of augmentation in the past (*n* = 15) or had a current history of augmentation (*n* = 11) (AUG). The median overall time between diagnosis of augmentation and testing was 23.36 weeks (IQR: 47.18–86.82). In seven of the 15 patients with a history of AUG the DA was stopped and alpha 2 delta ligands were started. In the remaining 8 patients the DA was decreased and/or switched to a long acting DA according to international guidelines.^4^ All patients with a history of AUG improved clinically after the DA was decreased or weaned off. Twenty‐seven patients, who did not show any signs of augmentation (RLS controls), were also included. All these patients were treated with dopaminergic medication compared to 66.5% of the AUG group, which was significantly different (see Table 1). There was no difference in age, disease duration, and scores on the International RLS Severity Scale (IRLS) between the AUG and the RLS control group. The 17 patients of the AUG group had a significantly higher LEDD than RLS controls (60 mg (36–90) vs. 36 mg (17.75–60); *P* = 0.012, Mann–Whitney *U* test) (Table 1).

**Table 1:** Demographics.

	HC	RLS controls	AUG	*P* value
Participants (*n*)	21	27	26	
Female (*n*,%)	15 (71.4)	19 (70.4)	17 (65.4)	0.887^a^
Age	59.20 (56.14–64.70)	62.92 (52.03–68.94)	64.85 (56.99–72.56)	0.162^b^
Education (y)	12 (11–14)	12 (11–13)	12 (10.25–12.00)	0.152^b^
Augmentation (*n*)				
Current	NA	NA	11	
Past	NA	NA	15	
Subthreshold (Current)	NA	NA	4 (2)	
Disease duration (y)	NA	12 (4.5–17.5)	15 (10–18)	0.095^c^
IRLS	NA	21.08 ± 7.59	21.42 ± 9.69	0.887^d^
LEDD (mg)	NA	36 (17.75–60)	60 (36–90)	**0.012** ^c^
Dopaminergic therapy (*n*, curr)	NA	27	17 (8)	**<0.001** ^a^
Pramipexole (curr)		18	8 (3)	
Ropinirole (curr)		1	1 (1)	
Rotigotine (curr)		5	7 (3)	
L‐Dopa (curr)		4	2 (2)	
ICD symptoms (*n*,%)	0	6 (22.2)	13 (50)	**0.047^e^**

Abbreviations: AUG, RLS patients with augmentation; curr, RLS patients with current augmentation; HC, Healthy controls; ICD, Impulsive compulsive disorder; IRLS, International Restless Legs Scale; LEDD, Levodopa equivalence daily dose; NA, Not applicable; *P*‐values numbers marked in bold indicate numbers that are significant; Quantitative values are given in mean ± SD or median and interquartile range; RLS, RLS patients without augmentation; Statistical methods: a: Chi‐squared test, b: Kruskal–Wallis test, c: Mann–Whitney *U* test, d: Student’s *t*‐test, e: Fisher’s Exact Test; y: Years.

ICD symptoms were significantly more common in AUG patients (*n* = 13, 50.0%) than in RLS controls (*n* = 6, 22.2%; *P* = 0.047, Fisher’s Exact Test).

There was an overall significant group difference in the anxiety subscale of the HADS (*P* = 0.021, Kruskal–Wallis test) with *η* ^2^ of 0.081 indicating a moderate effect size. Post hoc comparisons (Bonferroni corrected) showed a significance between RLS controls and HC (*P* = 0.026), but there were no other group differences (AUG vs. HC *P* = 0.094; RLS vs. AUG *P* = 1.000).

There was also a significant group difference in the depression scale of the HADS (*P* = 0.040, *η* ^2^ = 0.061, Kruskal–Wallis test). A post hoc analysis showed no significant difference between the AUG group and HC (*P* = 0.060), between RLS controls and HC (*P* = 0.100), or between the two patient groups (*P* = 1.000).

There was no significant difference between groups on the BIS‐11 (*P* = 0.089, ANOVA). However, when analyzing the attentional subdomain of the BIS‐11, we found a significant group difference (*P* = 0.015, *η* ^2^ = 0.135, ANOVA). Post hoc pairwise comparisons (Bonferroni corrected) revealed higher impulsivity scores for the AUG group than for HC (*P* = 0.015). There was no significant difference between RLS controls and HC (*P* = 0.122) or between RLS controls and AUG patients (*P* = 1.000).

There was a significant group difference in the mean total score of the TAS‐20 (*P* = 0.005, *η* ^2^ = 0.119, Kruskal–Wallis test) and in the subscores TAS‐F1 (*P* = 0.001, *η* ^2^ = 0.158, Kruskal–Wallis test) and TAS‐F2 (*P* = 0.011, *η* ^2^ = 0.099, Kruskal–Wallis test, see Table 2). Post hoc pairwise comparisons (Bonferroni corrected) revealed higher scores for the AUG group than for HC (*P* = 0.003). In contrast, there was neither a difference between HC and RLS controls (*P* = 0.192) nor between RLS controls and AUG patients (*P* = 0.520). None of the HCs, but six patients of the AUG group (23.1%) and 3 RLS controls (11.1%) scored above ≥61 points on the TAS‐20 (*P* = 0.056).

**Table 2:** Assessments grouped by augmentation.

	HC	RLS controls	AUG	*P* value
MMSE	30 (29–30)	29 (28–30)	29 (28.25–30)	0.452^b^
BIS‐11	59.90 ± 8.65	65.14 ± 9.20	65.05 ± 7.77	0.089^f^
BIS attentional	14.95 ± 3.89	17.05 ± 2.97	17.95 ± 2.70^†^	**0.015** ^f^
BIS motor	21.52 ± 3.09	21.86 ± 3.97	21.37 ± 3.56	0.905^f^
BIS nonplanning	23.43 ± 3.41	26.24 ± 4.36	25.74 ± 4.51	0.071^f^
Impulsive (BIS‐11 > 71) (*n*,%)	2 (9.5)	4 (19.0)	5 (26.3)	0.416^e^
HADS anxiety	5 (2–6)	8 (5.5–11)^‡^	6.5 (4–10.5)	**0.021** ^b^
HADS depression	3 (1–4)	4 (2.5–7.5)	4 (3–7)	**0.040** ^b^
TAS‐20	38 (32–43)	46 (31.5–55.5)	52 (39–58.75)^†^	**0.005** ^b^
TAS‐F1	10 (12–21.75)	14 (10–19)^‡^	15 (12–21.75)^†^	**0.001** ^b^
TAS‐F2	10 (5–12)	12 (8–15)	13.5 (10–16.75)^†^	**0.011** ^b^
TAS‐F3	17.95 ± 4.33	18.59 ± 6.22	20.65 ± 5.05	0.188^f^
Alexithymia (≥61 TAS‐20) (*n*,%)	0 (0)	3 (11.1)	6 (23.1)	0.056^e^
Emotion recognition (correct %)	76.2 (76.2–85.7)	76.2 (71.4–85.7)	66.7 (58.3–76.2)^†,¥^	**0.001** ^b^
FC Eye (n/s)	1.58 (1.37–1.84)	1.30 (0.77–1.97)	1.41 (1.09–1.64)	0.328^b^
FC Mouth (n/s)	0.67 ± 0.20	0.59 ± 0.21	0.50 ± 0.20^†^	**0.021** ^f^
FC Face (n/s)	2.92 ± 0.50	2.72 ± 0.72	2.69 ± 0.72	0.456^f^
TFD Eye (%)	42.8 ± 10.8	35.9 ± 16.2	39.7 ± 15.1	0.257^f^
TFD Mouth (%)	18.18 (13.42–28.79)	14.22 (11.29–26.86)	12.71 (10.46–19.36)	0.089^b^
TFD Face (%)	78.38 (74.87–83.65)	74.83 (68.20–80.91)	77.35 (67.50–83.13)	0.542^b^
TTFF Eye (msec)	586 (272–823)	657 (471–1432)	533 (346–1037	0.291^b^
TTFF Mouth (msec)	955 (605–1436)	1376 (885–2120)	1942 (1046–2267)^†^	**0.019** ^b^

*P*‐values numbers marked in bold indicate numbers that are significant; Quantitative values are given in mean ± SD for all data for better readability; RLS, RLS controls; s, Seconds; Statistical methods: a: Chi‐squared test, b: Kruskal–Wallis test, c: Mann–Whitney *U* test, d: Student’s *t*‐test, e: Fisher’s exact test, f: ANOVA; Statistical significant difference in the Bonferroni corrected post hoc pairwise comparison: ‡ between HC and RLS controls, †: between HC and AUG, ¥: between RLS controls and AUG; TAS: Toronto Alexithymia Scale; TTFF: Time to first fixation.

AUG, RLS patients with augmentation; BIS, Barratt Impulsiveness Scale; FC, Fixation count; FD, Fixation duration; HADS, Hospital Anxiety and Depression Scale; HC, Healthy controls; MMSE, Mini Mental State Examination; *n*, Number;

When analyzing the results of the eye tracking task, we found a significant group difference in the overall emotion recognition rate (*P* = 0.001, *η* ^2^ = 0.160, Kruskal–Wallis test). Post hoc analysis revealed a significant group difference between HC and the AUG group (*P* = 0.003) as well as between RLS controls and the AUG group (*P* = 0.009). There was no difference between HC and RLS controls (*P* = 1.000). Overall, HC and RLS controls recognized more emotions correctly than the AUG group.

When assessing the emotions separately, we found significant group differences for anger (*P* = 0.006, *η* ^2^ = 0.117, Kruskal–Wallis test), neutral (*P* = 0.009, *η* ^2^ = 0.106, Kruskal–Wallis test), and sadness (*P* = 0.018, *η* ^2^ = 0.086, Kruskal–Wallis test). The group difference for recognizing joy just missed significance (*P* = 0.058, Kruskal–Wallis test). After performing Bonferroni corrected post hoc analysis, the difference in recognizing neutral faces between the AUG group and HC (66.7% (66.7–100) vs. 100% (100–100), *P* = 0.029) as well as between the AUG group and RLS controls (100% (100–100), *P* = 0.045) reached significance. AUG patients performed less accurately than HC in anger and sadness trials (66.7% (33.3–66.7) vs. 100% (66.7–100), *P* = 0.007; 33.3% (33.3–66.7) vs. 66.7% (66.7–100), *P* = 0.002), but there was neither a difference between RLS patients with augmentation and RLS controls for anger (66.7% (66.7–100), *P* = 0.050) nor for sadness (66.7% (33.3–100), *P* = 0.075). There was no significant group difference for other emotions and no significant difference between HC and RLS controls (*P* > 0.8 in all analyses for separate emotions).

**Figure 1:**
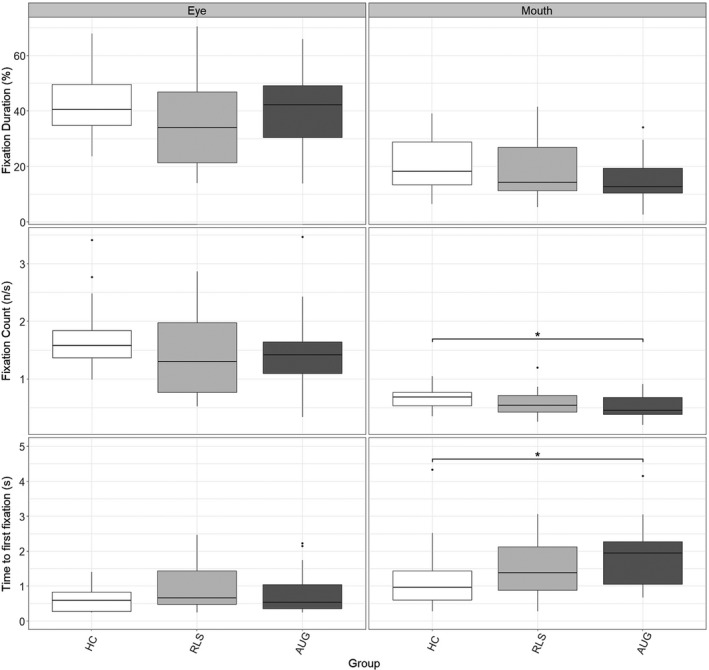
Eye tracking analysis. Abbreviations: HC, Healthy controls; RLS, RLS controls; AUG, RLS patients with augmentation; Outliers shown as dots; Significance shown with asterisk.

The analysis of eye movements revealed significant group differences in the relative fixation count (*P* = 0.021, *η* ^2^ = 0.106, ANOVA) and the time to first fixation in the mouth region (*P* = 0.019, *η* ^2^ = 0.083, Kruskal–Wallis test). Post hoc comparison (Bonferroni corrected) revealed AUG patients taking longer to look towards the mouth region (*P* = 0.022) than HC. Moreover, AUG patients had fewer fixations in the mouth region relative to HC (*P* = 0.018). There were no other relevant group differences (Fig. 1) for the predefined AOIs.

A Kendall’s tau correlation was run for the whole group to determine the relationship between TAS‐20 scores and emotion recognition rate (see Table 3). There was a weak, negative monotonic correlation between TAS‐20 and emotion recognition rate (*τ* = −0.169, *P* = 0.043). We also found a weak, negative monotonic correlation between the F2 subdomain of the TAS‐20 and the emotion recognition rate (*τ* = −0.172, *P* = 0.044). There was no correlation for the F1 and F3 subdomain of the TAS‐20.

**Table 3:** Kendall’s tau correlation analysis of emotion recognition rate; *P*‐values numbers marked in bold indicate numbers that are significant.

	Kendall’s tau	*Z* value	*P* value
TAS‐20	−0.16939	−2.02	**0.043**
TAS‐F1	−0.15790	−1.85	0.064
TAS‐F2	−0.17241	−2.01	**0.044**
TAS‐F3	−0.15966	−1.87	0.061

Finally, we compared AUG patients who scored positive on the QUIP with those who did not. There was no significant difference between AUG + ICD (*n* = 13) and AUG – ICD (*n* = 13) in terms of demographics, neuropsychological background scores, and other scales including TAS‐20 and eye tracking parameters (all *P* > 0.1).

There was no significant difference in HADS‐D (anxiety: *P* = 0.578, Welch’s *t*‐test; depression: *P* = 0.541, Welch’s *t*‐test), BIS‐11 (*P* = 0.461, Welch’s *t*‐test) or TAS‐20 (*P* = 0.094, Welch’s *t*‐test) between patients with current augmentation (*n* = 11) or those with a history of augmentation (*n* = 15). We found a group difference for the mean scores of the attentional subdomain of the BIS‐11 (current: 16.4 ± 1.51 vs. history: 18.8 ± 2.89; *P* = 0.029, *d* = −1.04, Welch’s *t*‐test) and in the F1 subdomain of the TAS‐20 (current: 12.9 ± 7.74 vs. history: 19.5 ± 6.24; *P* = 0.031, *d* = −0.942, Welch’s *t*‐test). There was no difference in the emotion recognition rate (*P* = 0.114, Welch’s *t*‐test) and only a trend for the time to first fixation in the mouth region (current: 2.228 ± 0.546 vs. history: 1.59 ± 0.99; *P* = 0.053, Welch’s *t*‐test). All other eye tracking parameters were not significant (all *P* > 0.1). However, these findings have to be handled with great caution, due to the small sample size and unequal group sizes.

## Discussion

In this study, we assessed both emotion recognition and alexithymia in RLS patients with augmentation. We found that RLS patients with augmentation (past or current) showed higher mean total scores on the TAS‐20, a psychometric measure for alexithymia, than healthy controls. The scores were similar compared to previously published reports in patients with Parkinson’s disease with established ICDs.^17^

It is worth noting that only few patients and none of the healthy controls fulfilled the established criteria for definitive alexithymia (total score ≥ 61), with significantly more RLS patients with than without augmentation. However, the mean scores in the AUG group (>50) almost reached the established cut off score for borderline alexithymia (≥52), and borderline alexithymia scores were previously linked with emotion dysregulation, impulsivity, and aggression.^33^ Furthermore, and in contrast to a previous study,^34^ we did not find a significant difference on the TAS‐20 scores between RLS patients without augmentation and healthy controls. It is possible that a subgroup of patients in the previous study by Yilmaz et al.^34^ had augmentation or ICD symptoms which may have influenced their results.

Patients with augmentation had an impairment in facial emotion recognition, particularly toward negative emotions (anger, sadness). The difference for the positive emotion joy just missed significance. It is likely that a larger sample size or other positive emotions, which may be harder to recognize, would have also revealed a significant impairment in the RLS group with augmentation.

Previous studies linked alexithymia with impaired emotion recognition^35^, ^36^, ^37^, ^38^ and in PD ICDs with a reduced bias towards negative and positive emotions compared to healthy controls.^39^ Results of this study are in line with these previous findings showing that individuals with high alexithymia scores have an impaired ability to identify emotions correctly.

Interestingly, we found that RLS patients with augmentation took longer to look towards the mouth area and had also fewer fixations in this region compared to healthy controls. A previous study has shown that particularly the mouth area is important to discriminate basic facial expressions^40^ which may be responsible for the impaired emotion recognition in the augmentation group.

In line with our previous results,^5^ we found ICD symptoms more frequently in RLS patients with augmentation in this study. Patients with augmentation show multiple traits that have been linked to impulsivity such as alexithymia and impaired face emotion recognition. Our results strengthen further the hypothesis that augmentation and impulsivity may share a common pathophysiology in RLS.

There are, however, also potential limitations. Although the majority of RLS patients with augmentation had dopaminergic therapy, the DA medication was already weaned off in 9 individuals. At the time of the investigation, these nine patients were treated with alpha 2 delta ligands according to the recommended guidelines.^4^ The time between clinical evaluation of augmentation and when eye tracking was performed could potentially influence the outcome of assessments. Furthermore, only about half of the patients had augmentation at the time of testing, whereas the rest of the patients in the augmentation group did not have augmentation at the time of testing. However, it has been shown that neuropsychiatric changes persist, albeit in patients with substance abuse even after prolonged abstinence^41^ and in patients with Parkinson’s disease after withdrawal of DA.^42^ Moreover, several studies have reported cortico‐striatal changes as a result of chronic DA use.^7^, ^43^ Therefore, we hypothesize that DA induce long‐term neuroplastic changes in vulnerable patients with RLS, which may cause impulsivity and poorer emotional recognition even when the DA are already weaned off. Furthermore, we have not specifically assessed sleep quality in our study sample, which may have influenced the results. However, the role of sleep deprivation and emotional recognition remains unclear^44^ and was mostly evident in tasks using morphed faces (complex tasks)^45^ which was not the case in this study. Moreover, we did not use the Augmentation Severity Rating Scale to assess severity of augmentation. Finally, the sample size of our study is small and therefore larger studies are needed to confirm our findings.

In conclusion, we found impaired emotional processing in RLS patients with augmentation compared to RLS controls and healthy controls. Furthermore, we found that RLS patients with augmentation had a longer time to first fixation and a lower fixation count in the mouth region. It is possible that the altered facial exploration pattern is responsible for the poorer emotional discrimination.

## Conflict of Interest

None declared.
